# Combining Dextran Conjugates with Stimuli-Responsive and Folate-Targeting Activity: A New Class of Multifunctional Nanoparticles for Cancer Therapy

**DOI:** 10.3390/nano11051108

**Published:** 2021-04-25

**Authors:** Manuela Curcio, Alessandro Paolì, Giuseppe Cirillo, Sebastiano Di Pietro, Martina Forestiero, Francesca Giordano, Loredana Mauro, Diana Amantea, Valeria Di Bussolo, Fiore Pasquale Nicoletta, Francesca Iemma

**Affiliations:** 1 Department of Pharmacy, Health and Nutritional Sciences, University of Calabria, 87036 Rende, Italy; manuela.curcio@unical.it (M.C.); alessandro.paoli28@gmail.com (A.P.); giuseppe.cirillo@unical.it (G.C.); marti.forestiero@libero.it (M.F.); francesca.giordano@unical.it (F.G.); loredana.mauro@unical.it (L.M.); diana.amantea@unical.it (D.A.); francesca.iemma@unical.it (F.I.); 2 Department of Pharmacy, University of Pisa, Via Bonanno Pisano 33, 56126 Pisa, Italy; sebastiano.dipietro@unipi.it (S.D.P.); valeria.dibussolo@unipi.it (V.D.B.)

**Keywords:** dextran conjugate, folic acid, pH/redox responsive nanoparticles, targeted release, cystamine, PEG diacid

## Abstract

Nanoparticles with active-targeting and stimuli-responsive behavior are a promising class of engineered materials able to recognize the site of cancer disease, targeting the drug release and limiting side effects in the healthy organs. In this work, new dual pH/redox-responsive nanoparticles with affinity for folate receptors were prepared by the combination of two amphiphilic dextran (DEX) derivatives. DEXFA conjugate was obtained by covalent coupling of the polysaccharide with folic acid (FA), whereas DEXssPEGCOOH derived from a reductive amination step of DEX was followed by condensation with polyethylene glycol 600. After self-assembling, nanoparticles with a mean size of 50 nm, able to be destabilized in acidic pH and reducing media, were obtained. Doxorubicin was loaded during the self-assembling process, and the release experiments showed the ability of the proposed system to modulate the drug release in response to different pH and redox conditions. Finally, the viability and uptake experiments on healthy (MCF-10A) and metastatic cancer (MDA-MB-231) cells proved the potential applicability of the proposed system as a new drug vector in cancer therapy.

## 1. Introduction

In the last decades, the acquisition of ever more complete information about the physiopathological features of cancer tissues coupled with the tremendous progresses in nanotechnology applied in the biomedical field, and in cancer therapy in particular, has led to the development of several nanoparticles for the targeted release of anticancer drugs as an alternative approach to overcome the well-known limits of conventional chemotherapy [1,2].

By virtue of the Enhanced Permeation and Retention (EPR) effect, a typical condition of the tumor tissues characterized by angiogenesis and lack of lymphatic drainage, nanoparticles can efficiently accumulate at the tumor site [3,4]. In addition, this kind of system can be designed to recognize specific elements in cancer cells (i.e., overexpressed membrane receptors) [5,6] and/or to respond to specific signals (variation of temperature, pH, redox potential) from the tumor microenvironment [7], enhancing, in both cases, the amount of drug released in the target site. More in detail, it is well known that folate receptors are overexpressed in many solid tumors [8,9], that the extracellular environment is more acidic (pH 6.5) in tumors than in blood and in normal tissues (pH 7.4) [10,11], and that pH values of endosomes/lysosomes are even lower (5.0–5.5) [12,13]. Moreover, the glutathione (GSH) concentration in cancer cells (approximately 2–10 mM) is almost 1000-fold higher than the extracellular matrix (approximately 2–20 μM), generating a high redox potential across cell membranes [14]. Taken together, all these pieces of evidence can serve as ideal triggers for the vectorization of anticancer drugs in tumor cells mediated by nanoparticle systems [15].

Natural polymers, and polysaccharides in particular, were extensively investigated as base materials for the preparation of targeted nanocarriers because of their non-toxicity, cost-effectivity, and physico-chemical features [16,17].

Among others, dextran (DEX), a bacterial-deriving glucose homopolysaccharide, is widely employed in drug delivery due to its high-water solubility, biocompatibility, biodegradability, resistance to protein adsorption, and ease of chemical modification due to the presence of reactive hydroxyl groups [18,19,20].

The functionalization of DEX with hydrophobic moieties endowed with targeting activity is a useful approach to obtain self-assembling nanocarriers able to vectorize the payload in cancer cells [21]. As an example, the derivatization of DEX with folic acid (FA) generated actively targeted nanoparticle structures for the release of Doxorubicin (DOX) in breast cancer [22,23]. Similarly, the conjugation with hydrophobic chemical species endowed with disulfide bridges, hydrazone, or imine bonds carried out to self-assembling materials for the pH and redox responsive release of anticancer drugs [18,24,25].

In this work, we prepared two new amphiphilic DEX derivatives, DEXFA and DEXssPEGCOOH, with targeted and stimuli-responsive activity, respectively. DEXFA conjugate derived from the covalent coupling of the polysaccharide with FA, whereas DEXssPEGCOOH was obtained from the reaction of a cystamine-modified DEX with polyethylene glycol 600 diacid (PEG_600_COOH). The combination of both derivatives allowed obtaining multifunctional self-assembling nanoparticles (DFNPs) with active-targeted and pH/redox responsive activities. The nanoparticles were characterized by Dynamic Light Scattering (DLS) and Transmission Electron Microscopy (TEM), whereas pH/redox-triggered destabilization assays were performed by measuring the variation of the nanoparticles mean diameter in reducing media and acidic pH. DFNPs were loaded with Doxorubicin hydrochloride (DOX), a DNA topoisomerase II inhibitor with a broad-spectrum antineoplastic activity [26], and in vitro release experiments from DOX-loaded DFNPs were performed varying the pH and redox potential of the surrounding medium. Cytotoxicity and cellular uptake experiments were performed on healthy (MCF-10A) and cancer (MDA-MB-231) cells to evaluate the safety and potential suitability of the system in cancer therapy. Ultimately, cell cycle analysis confirmed the efficacy of the drug delivery system tested in MDA-MB-231 cells.

## 2. Materials and Methods

### 2.1. Synthesis of DEXcys

DEXcys was obtained by reductive amination [27,28]. Briefly, after 0.2 g (1.2 mmol glucose repeating units) of DEX (40 kDa) was dissolved in 20 mL of a H_2_O:DMSO (3:7 *v*/*v*) mixture, 0.78 g (12.3 mmol) of sodium cyanoborohydride and 2.78 g (12.3 mmol) of cystamine dihydrochloride (cysHCl) were added, and the mixture was stirred for 24 h at room temperature. The resulting solution was purified by dialysis (MWCO 12–14 kDa) against water at 20 °C for 72 h and finally freeze-dried (98% yield). ^1^H-NMR and 2D-HSQC spectra were recorded on a Bruker Avance III 400 MHz (Bruker Italy, Milan, Italy) at 25 °C using a DMSO/D_2_O (1:1 *v*/*v*) mixture as solvent. Dialysis membranes were purchased from Medicell International LTD (London, UK).

All chemicals were purchased from Merck/Sigma Aldrich, Darmstadt, Germany.

### 2.2. Synthesis of DEXssPEGCOOH Conjugate

PEG_600_diacid (0.063 g), 1-etil-3-(3-dimetilamminopropil) carbodiimide (EDC) (0.04 g, 0.21 mmol), and *N*-hydroxy succinimide (0.024 g, 0.21 mmol) were dissolved in 3 mL of DMSO and left to react for 1 h at room temperature under magnetic stirring. Then, DEXcys (0.017 g) dissolved in 2 mL DMSO was added. The mixture was magnetically stirred for 24 h at room temperature, purified by dialysis (MWCO 12–14 kDa) against water at 20 °C for 72 h, and finally freeze-dried (98% yield). Dialysis membranes were purchased from Medicell International LTD (London, UK). ^1^H-NMR and 2D-HSQC spectra were recorded on a Bruker Avance III 400 MHz (Bruker Italy, Milan, Italy) at 25 °C using DMSO/D_2_O (1:1 *v*/*v*) mixture as solvent. All chemicals were purchased from Merck/Sigma Aldrich, Darmstadt, Germany.

### 2.3. Synthesis of DEXFA

FA (0.5 g, 1.13 mmol) was dissolved in 10 mL DMSO, then 0.66 g (3.44 mmol) of EDC, 0.44 g (3.47 mmol) of NHS, and 0.5 g (2.98 mmol) of DEX were added, and the mixture was left to react for 48 h at 45 °C under magnetic stirring. The resulting solution was purified by dialysis (MWCO 12–14 kDa) against a phosphate buffer (0.01 M, pH 7.4) and water for 24 and 48 h, respectively, and finally freeze-dried (98% yield). ^1^H-NMR and 2D-HSQC spectra were recorded on a Bruker Avance III 400 MHz (Bruker Italy, Milan, Italy) at 25 °C using DMSO as solvent. Dialysis membranes were purchased from Medicell International LTD (London, UK). All chemicals were purchased from Merck/Sigma Aldrich, Darmstadt, Germany.

### 2.4. Determination of the Critical Aggregation Concentration (CAC)

The CAC of DEXssPEGCOOH, DEXFA, and their combination in the aqueous phase were measured by fluorescence analysis using pyrene as a nonpolar probe [29,30]. In separate experiments, 20.0 μL pyrene solution at a concentration of 3.0 × 10^−5^ M in acetone was evaporated in vials. Meanwhile, each conjugate was dissolved at concentrations ranging from 1.6 × 10^−7^ to 1 mg mL^−1^ in phosphate buffer (0.01 M, pH 7.4) under magnetic stirring, and 1 mL of each solution was added to the pyrene vials. The content of the vials was mixed for 12 h, thereby leading to solutions with pyrene concentration of ca. 6.0 × 10^−7^ M. Then, the intensity ratios (I_3_/I_1_) of the third vibronic band at 385 nm to the first one at 373 nm of the fluorescence emission spectra of pyrene were recorded at 25 °C. Pyrene fluorescence emission spectra (λ_exc_ = 336 nm; λ_em_ = 350–500 nm) were recorded on Hitachi F-2500 spectrometer (Tokyo, Japan). All chemicals were purchased from Merck/Sigma Aldrich, Darmstadt, Germany.

### 2.5. Preparation of Nanoparticles and DOX Loading

In the same vial, DEXssPEGCOOH and DEXFA were dispersed in phosphate buffer solution (0.01 M, pH 7.4) at a final concentration of 1 mg mL^−1^ and magnetically stirred for 2 h at 25 °C. DOX-loaded nanoparticles (DOX@DFNPs) were prepared by dispersing each conjugate (final concentration 1 mg mL^−1^) in a 58.8 μM DOX hydrochloride solution in phosphate buffer (0.01 M, pH 7.4) and magnetically stirred for 12 h at room temperature [31]. The dispersion was used as such in the next release experiments. The DOX content in DOX@DFNPs was confirmed by diluting 1 mL of DOX@DFNPs dispersion in 25 mL of methanol, in order to disrupt nanoparticle structure [32], followed by the measurement of the fluorescence of the solution (λ_exc_ = 480 nm; λ_em_ = 590 nm).

Size distributions were determined using a 90 Plus Particle Size Analyzer DLS equipment (Brookhaven Instruments Corporation, New York, NY, USA) at 25 °C. The autocorrelation function was measured at 90° and the laser beam operated at 658 nm. The polydispersity index (PDI) was directly obtained from the instrumental data fitting procedures by the inverse Laplace transformation and Contin methods. PDI values ≤ 0.3 indicate homogeneous and mono-disperse populations [33]. Morphological analysis of nanoparticles was carried out using transmission electron microscopy (TEM; HRTEM/Tecnai F30 [80 kV] FEI company, Hillsboro, OR, USA). A drop of the vesicle dispersion was placed on a Cu TEM grid (200 mesh, Plano GmbH, Wetzlar, Germany), and the sample in excess was removed using a piece of filter paper. A drop of 2% (*w*/*v*) phosphotungstic acid solution was then deposited on the carbon grid for 2 min. Once the excess of staining agent was removed with filter paper, the samples were air-dried, and the thin film of stained nanoparticles was observed.

### 2.6. Destabilization Experiments

Destabilization experiments of empty nanoparticles in reductive environments were performed by the dialysis method. Briefly, in separate experiments, 4 mL of freshly prepared empty nanoparticles (final concentration 1 mg mL^−1^) were loaded in a dialysis bag (MWCO 12–14 kDa) and dialyzed against 30 mL phosphate (0.01 M, pH 7.4) and an acetate buffer (0.01 M, pH 5.5) containing GSH at different concentrations (0 and 10 mM) at 37 °C in a beaker with constant stirring. After 24 h, the mean diameter and PDI were measured by DLS. Each analysis was performed in triplicate.

### 2.7. Release Experiments

Release experiments were carried out by means of a dialysis method under sink conditions. In two different experiments, 2 mL DOX@DFNPs dispersion were loaded in a dialysis bag (cut-off molecular weight of 3.5 kDa) and dialyzed against 10 mL phosphate (0.01 M, pH 7.4) and an acetate (0.01 M, pH 5.5) buffer containing GSH at different concentrations (0 and 10 mM) at 37 °C in a beaker with constant stirring. At pre-established times, samples (0.5 mL) of release medium were withdrawn, replaced with fresh medium, and quantified by a fluorescence spectrometer (F-2500 Hitaki, Tokyo, Japan). Experiments were performed in triplicate.

### 2.8. Stability in Plasma Simulating Medium

The stability of DOX@DFNPs dispersion in plasma simulating fluid was performed by measuring the drug content in PBS solution containing 90% FBS [34]. In particular, 2 mL DOX@DFNPs dispersion was inserted in a dialysis bag (cut-off molecular weight of 3.5 kDa) and dialyzed against 10 mL of phosphate buffer solution (0.01 M, pH 7.4) containing 90% FBS at 37 °C in a beaker with constant stirring. At pre-established times, samples (0.5 mL) of release medium were withdrawn, replaced with fresh medium, and quantified by a fluorescence spectrometer (F-2500 Hitaki, Tokyo, Japan). Experiments were performed in triplicate.

### 2.9. Cell Culture

MCF-10A and MDA-MB-231 cell lines were from American Type Culture Collection (Manassas, VA, USA). All cell lines were authenticated and stored according to the supplier’s instructions. Cells were used within four months after recovery of frozen aliquots and regularly tested for mycoplasma-negativity (MycoAlert Mycoplasma Detection Assay, Lonza, Basel, Switzerland). The MCF-10A cell line, a non-tumorigenic human epithelial breast cell line, was cultured in DMEM/F-12 supplemented with 5% horse serum (HS), L-glutamine (1%), penicillin/streptomycin (1%), 100 ng mL^−1^ cholera toxin, hydrocortisone (0.5 mg mL^−1^), insulin (10 mg mL^−1^), and epidermal growth factor (EGF) (20 ng mL^−1^). MDA-MB-231 human breast cancer cells were cultured in Dulbecco’s modified Eagle’s medium (DMEM)/Nutrient Mixture F-12 Ham (DMEM/F12) supplemented with 5% fetal bovine serum (FBS) containing L-glutamine (1%) and penicillin/streptomycin (1%). The cells were maintained at 37 °C in a 5% CO_2_ humidified incubator. Before each experiment, cells were grown in a phenol red-free medium, containing 5% charcoal-stripped FBS (cs-FBS), for at least 24 h.

### 2.10. Cell Viability Assay

The effect of DOX, DFNPs, and DOX@DFNPs was tested in MCF-10A and MDA-MB-231 cells using the 3-(4,5-dimethylthiazol-2-yl)-2,5-diphenyltetrazolium bromide (MTT) cell viability assay. Cells were plated in the appropriate medium (1 × 10^4^) in 96-well tissue culture plates and incubated for 24 h at 37 °C and 5% CO_2_, to allow cell adhesion. After 24 h, the culture medium was replaced with medium supplemented with 5% cs-FBS, and the cells were treated with DOX (0.08–1.28 μg mL^−1^) and/or DFNPs (0.625–1.0 mg mL^−1^) for 72 h. The MTT assay was performed adding 100 µL MTT stock solution in PBS (2 mg mL^−1^) to each well and incubated for 4 h to allow the formation of violet formazan crystals. Then, the culture medium was removed, and 100 µL of DMSO was added to solubilize the formazan crystals. The absorbance was measured with the Multiskan EX Microplate Reader (Thermo Scientific, Waltham, MA, USA) at the wavelength of 570 nm.

### 2.11. Cellular Uptake Experiments

MDA-MB-231 and MCF-10A cells were treated or untreated with DOX and DOX@DFNPs for 24 h. After incubation, the cells were washed twice with phosphate-buffered saline (PBS, pH 7.4) and fixed in 3.7% formaldehyde solution in PBS for 10 min at room temperature and then washed again twice in PBS. The cells were permeabilized with a solution of 0.1% Triton X-100 in PBS for 3–5 min, followed by two washes in PBS. 4′,6-diamidino-2-phenylindole dihydrochloride (DAPI, 2 g mL^−1^) staining was used for nuclei detection. To confirm the folate receptor-mediated uptake efficacy of DOX@DFNPs, both cell lines were pretreated with free FA (20 μg) for 1 h [35]. The cellular uptake of DOX and DOX@DFNPs (excitation 480 nm, emission 590–610 nm), was quantified in MDA-MB-231 cells using a confocal laser scanning microscope (Fluoview FV300, Olympus, London, UK). Fluorescence intensity was measured in three different (40×) optic fields of three different wells per each experimental condition.

### 2.12. DNA Flow Cytometry

To perform cell cycle analysis, MDA-MB-231 cells were treated or untreated with DOX or DOX@DFNPs for 48 h. The cells were then fixed with a solution of propidium iodide (100 µg mL^−1^), and then RNase A (20 µg mL^−1^) was added. Cellular cycle was measured using a FACScan flow cytometer (Becton Dickinson, Mountain View, CA, USA) and the data acquired using CellQuest software. Cell cycle profiles were determined using Mod-Fit LT.

## 3. Results and Discussion

The design of drug delivery systems able to recognize characteristic elements or respond to specific signals of the tumor tissues is the main strategy to target the drug release to the site of interest, optimizing the drug efficacy and minimizing the insurgence of undesirable side-effects. In this work, two different self-assembling specimens endowed with actively targeted (DEXFA) and pH/redox-responsive (DEXssPEGCOOH) elements were combined to obtain the DFNPs nanoparticles with affinity to folate receptors (through folate moieties) and the ability to trigger the drug release in response to different pH and GSH concentrations due to the presence of COOH functionalities and cystamine residues, respectively.

### 3.1. Synthesis and Characterization of DEXssPEGCOOH

**Figure 1:**
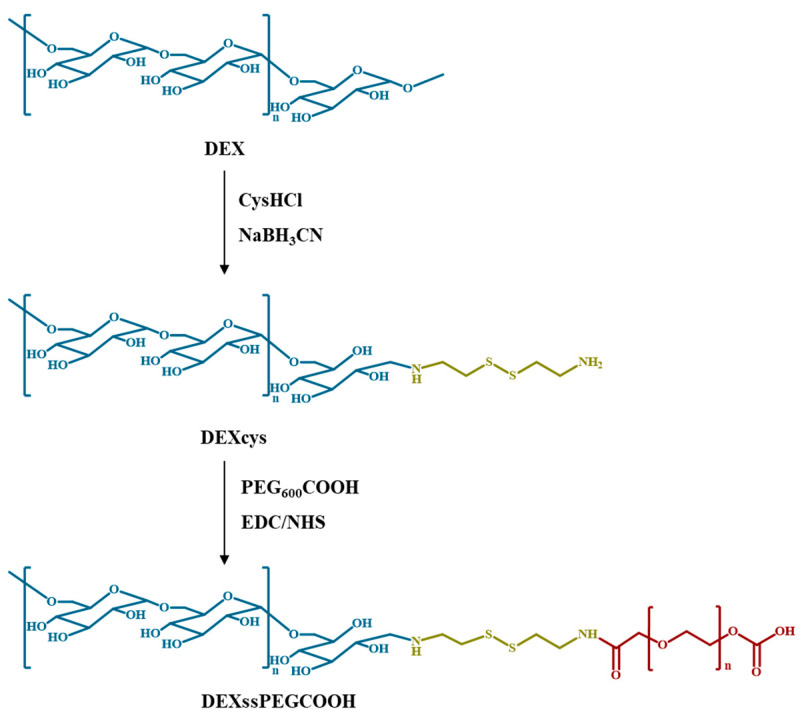
Synthesis of DEXcys and DEXssPEGCOOH.

As depicted in Figure 1, DEXssPEGCOOH conjugate was obtained by a two-step procedure consisting of (i) reductive amination of DEX in presence of cysHCl and sodiumcyano-borohydride and (ii) covalent coupling of DEXcys and PEG_600_COOH via carbodiimide chemistry.

**Figure 2:**
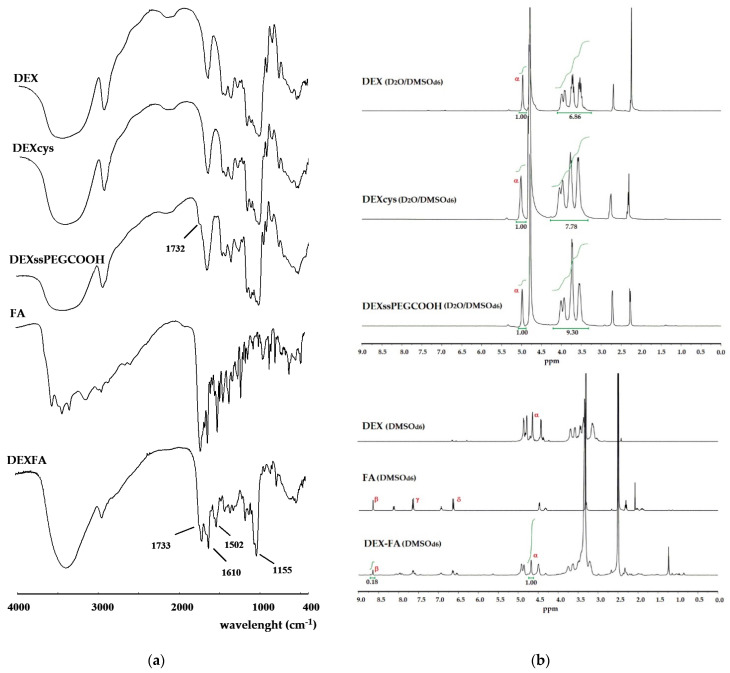
(**a**) FT-IR and (**b**) ^1^H-NMR spectra of DEX and DEX conjugates.

DEXcys and DEXssPEGCOOH were characterized by FT-IR and ^1^H-NMR analyses (Figure 2). In FT-IR spectrum of DEXcys (Figure 2a), no significant variation of the absorption bands was observed compared to native DEX, because the signals of cystamine were overshadowed by the more intense absorption bands of the polysaccharide. In the ^1^H-NMR spectrum (Figure 2b), the signal of DEX anomeric proton (β) was evident at around 4.9 ppm, while the presence of cystamine residues was confirmed by the enhancement of the relative intensity of aliphatic methylene protons in the region from 3.1 to 4.2 ppm with respect to DEX spectrum. In particular, from the ratio between the intensity of aliphatic to anomeric protons of DEX and DEXcys, a derivatization degree of 9.2% was estimated. The successful covalent attachment of PEG_600_COOH on DEX was confirmed by the appearance, in FT-IR spectrum (Figure 2a), of new absorption bands at 1732 cm^−1^, ascribable to C=O stretching of carboxyl functions, respectively. The further increase of the aliphatic region in ^1^H-NMR spectrum of DEXssPEGCOOH conjugate allowed estimating that a 38% amount of cystamine residues in DEXcys was coupled with PEG_600_COOH.

For both conjugates, 2D-HSQC spectra were recorded to further characterize the system (Figures S1 and S2 in Supplementary Material). DEXcys spectrum showed a 64 ppm resonance signal, ascribable to the cystamine protons, in addition to that attributed to the dextran carbon backbone, whereas in DEXssPEGCOOH conjugate an additional 70 ppm resonance, due to the -O-CH_2_CH_2_-O- repetitive PEG fragment, was observed.

In order to achieve further information about the conjugates structures, their diffusion coefficients were measured by ^1^H-DOSY analyses [36] (see Supplementary Materials). A slight decrease of the diffusion coefficients was found moving from DEX (1.60 × 10^−10^ m^2^/s), DEXcys (1.52 × 10^−10^ m^2^/s), and DEXssPEGCOOH (1.33 × 10^−10^ m^2^/s), as a consequence of the functionalization process enhancing the molecular weight of the resulting conjugate (Figures S4–S6 in Supplementary Materials). In addition, the DOSY spectra can be rationalized as a further confirmation of the purity of the conjugates: no small molecule signal was envisaged superimposed under the polymer proton resonances, with the exponential fittings of the decay curves for the different signals of the proton spectrum obtained with very small errors, and in line with each other (Figures S5 and S6 in Supplementary Materials).

### 3.2. Synthesis and Characterization of DEXFA Conjugate

**Figure 3:**
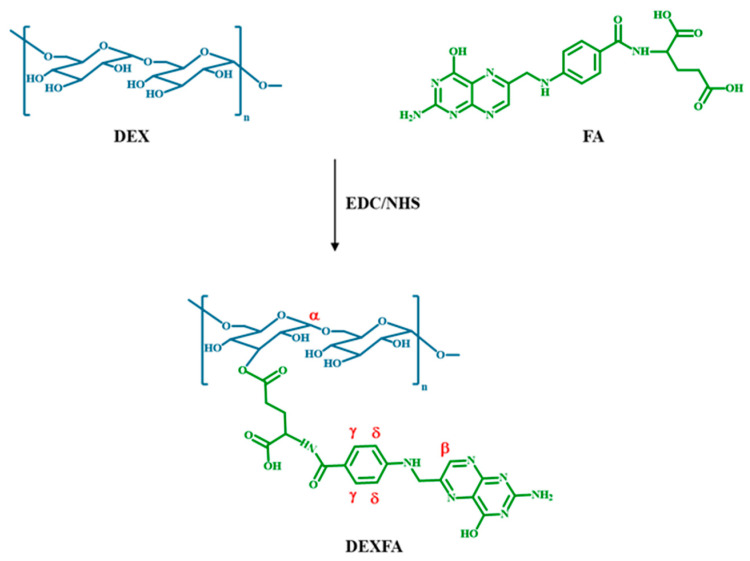
Synthesis of DEXFA conjugate.

DEXFA conjugate was prepared through esterification reaction between the γ-carboxylic acid group of FA and the hydroxyl groups of DEX (Figure 3).

In the FT-IR spectrum of DEXFA (Figure 2a), new absorption bands at 1733 and 1610 cm^−1^, ascribable to C=O and -C=C- stretching vibration of FA, respectively, were observed. In addition, the characteristic bands of -NH_2_ and -CONH- stretching vibrations at 1502 and 1155 cm^−1^ indicated the successful grafting of FA onto DEX. In the ^1^H-NMR spectrum of DEXFA (Figure 2b), the signal of the DEX anomeric proton (α) was observed at 4.68 ppm, while the presence of FA residues was evidenced by the characteristic resonance signals of FA at 8.5 (pteridine proton, β), 7.6, and 6.8 ppm (γ and δ aromatic protons). The relative integration of pteridine proton peak (β) and anomeric proton (α) allowed calculating a derivatization degree (DD) of 18%, expressed as FA moieties with respect to DEX repeating units. Additionally, for DEXFA, a 2D-HSQC spectrum (Figure S3 in Supplementary Materials) was recorded, and it is in accordance with the reported proton spectrum.

### 3.3. Determination of CAC of DEXssPEGCOOH and DEXFA

**Figure 4:**
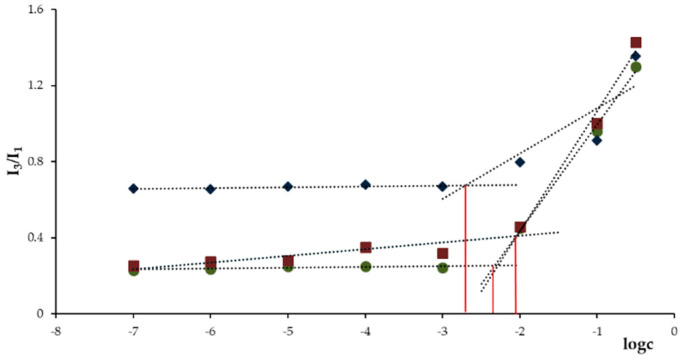
Dependence of pyrene fluorescence spectrum signals on (●) DEXFA, (♦) DEXssPEGCOOH, and (■) DEXFA/DEXssPEGCOOH concentration at pH 7.4.

The amphiphilic properties of DEXssPEGCOOH, DEXFA, and the combination of two conjugates were evaluated by measuring their CAC, defined as the concentration value above which the conjugates can organize in micellar structures. The determination of this parameter is crucial in view of a potential application in vivo, where the system is highly diluted in the systemic circulation: the lower the CAC value, the greater the stability of the micellar structures formed at low conjugate concentrations. For this measurement, pyrene was used as a probe in virtue of the ability to modify its fluorescent properties when located inside or in the proximity of the micellar hydrophobic domains. In Figure 4, the dependence of pyrene fluorescence spectra (I_385_/I_373_ ratio) on the logarithm of conjugate concentration was reported. At low concentrations, the intensity values remained almost unchanged; in contrast, when the amount of conjugate increased, a sharp change of the intensity was observed, indicating the onset of self-assembly. From the crossover points, CAC values of 1.66 and 5.60 μg mL^−1^ were calculated for DEXssPEGCOOH and DEXFA, respectively. The lower CAC of DEXssPEGCOOH could be ascribable to the amphiphilic behavior of PEG enhancing the ability of the conjugate to self-assembly in water media. When the experiments were performed on the combination of the two DEX conjugates, in according with literature data [37], a further increase of the CAC value was recorded (8.9 μg mL^−1^) as a consequence of the enhanced solubility of the whole system.

### 3.4. Preparation of DFNPs

**Figure 5:**
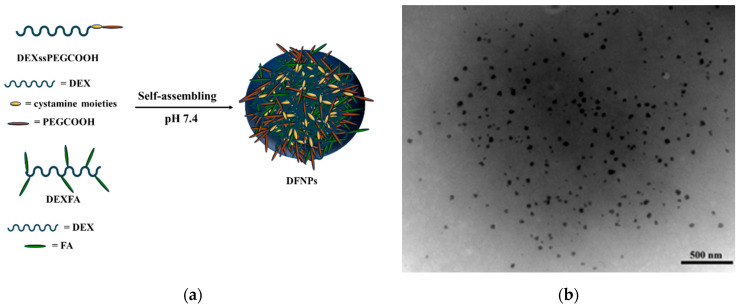
(**a**) Representation of self-assembling process and (**b**) TEM image of DFNPs.

DFNPs were prepared in a straightforward procedure exploiting the amphiphilic character of the systems by dispersing the DEXssPEGCOOH and DEXFA conjugates in phosphate buffer solution at pH 7.4 (Figure 5).

We can hypothesize that FA and PEG moieties in DEXFA and DEXssPEGCOOH conjugates constitute the hydrophobic core of the system, respectively, while the polysaccharide represents the hydrophilic nanoparticle corona.

DLS analysis showed a unimodal particle size distribution, with a mean hydrodynamic diameter of 50 ± 5 nm and a PDI of 0.1, while TEM micrographs revealed that the DFNPs were characterized by spherical shape and a mean diameter value close to that recorded by DLS (Figure 5b).

DFNPs present an ideal size to accumulate in tumor tissues via the EPR effect and allow an extensive tumor penetration [38].

### 3.5. Destabilization Experiments

The main feature of a stimuli-responsive nanocarrier is the ability to undergo specific structural modifications in response to internal or external stimuli in order to enhance cellular internalization and drug release in the diseased site, while remaining stable in the systemic circulation [39,40]. Due to the presence of pH and redox-responsive functionalities, it is expected that DFNPs can be destabilized, varying the pH and the GSH concentration in the surrounding medium. For this determination, the nanoparticles size changes in different pH and redox conditions were evaluated by DLS analyses after 24 h incubation.

In phosphate buffer pH 7.4, mimicking the extracellular environment, the mean diameter of DFNPs remained almost unchanged, while, when 10 mM GSH was added, the mean particle size and the PDI increased to 90 nm and 0.41, respectively, because of the breakage of crosslinking points (disulfide bonds) in the nanoparticle structure [41]. At pH 5.0 (close to the pKa value of PEGCOOH moieties of DEXssPEGCOOH conjugate), the particle diameter rose to 150 nm, probably as a consequence of the nanoparticle aggregation in larger structures due to the formation of intermolecular hydrogen bonds [42]. Finally, the incubation of the nanoparticles in acetate buffer pH 5.0 containing 10 mM GSH resulted in a further increase of the mean diameter and PDI value (nearly to 170 nm and 0.42, respectively), as a consequence of the simultaneous breakage of disulfide bonds and the aggregation phenomena that carried out to the destabilization of the nanoparticles.

### 3.6. DOX Loading and Release Experiments

**Figure 6:**
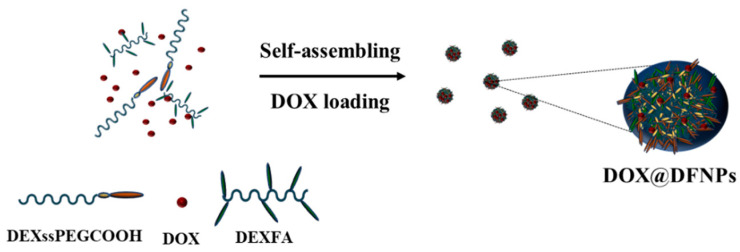
Schematization of DOX loading procedure.

DOX hydrochloride was loaded into the nanoparticles in a one-step procedure, adding the drug at a concentration of 32 mg per g of conjugate during the self-assembling of the conjugates (Figure 6), obtaining DOX@DFNPs systems showing similar morphological and dimensional behavior of the unloaded DFNPs. DOX@DFNPs dispersion was used as such in the release experiments, assuming that the total amount of added DOX was absorbed by the nanoparticles.

**Figure 7:**
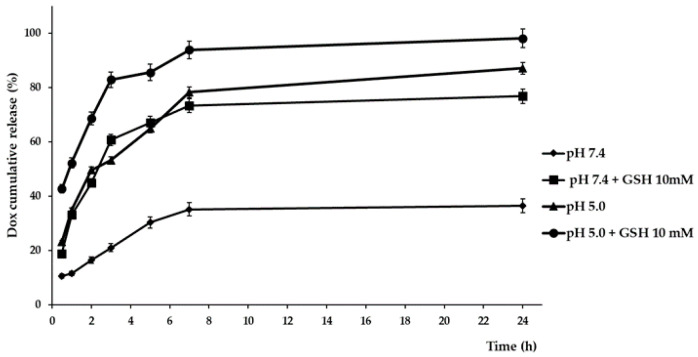
DOX release profiles from DOX@DFNPs in different pH and redox conditions.

Release experiments from DOX@DFNPs were performed in media mimicking the extracellular (phosphate buffer at pH 7.4) and intracellular (acetate buffer at pH 5.0) environments with or without GSH 10 mM (Figure 7).

At pH 7.4, simulating normal physiological conditions, a controlled release profile, not exceeding 36% in the first 24 h, was recorded, indicating that the nanoparticles were stable in the extracellular medium of healthy tissues and only a small amount of drug would be leaked during blood circulation.

In pH 7.4 medium containing 10 mM GSH and in acetate buffer at pH 5.0, similar release profiles were recorded, with percentages near to 35% and 80% in the first 60 min and 24 h, respectively. The data, in accordance with the stability experiments, are a consequence of the destabilization of the nanoparticle structure due, in the case of GSH in phosphate buffer, to the breakage of disulfide bonds, and in the case of acetate buffer at pH 5.0, to the modification of the ionization state of both DFNPs and drug. These phenomena modified the drug to polymer interactions, enhancing the drug release. Finally, when GSH was added to acetate buffer, the highest release percentages were observed at all the experimental times (52% and 98% after 60 min and 24 h, respectively), demonstrating the synergistic effect of pH and redox stimuli on the in vitro drug release and the suitability of the proposed system as redox/pH responsive drug delivery device. The stability of the loaded nanoparticles in plasma-simulating fluid was also performed using an FBS-containing phosphate buffer solution as the release medium. As expected, a controlled release profile very similar to that observed in the phosphate buffer in absence of FBS was recorded, with the nanoparticles maintaining more than 70% of the drug loaded after 24 h.

### 3.7. Biological Characterization

**Figure 8:**
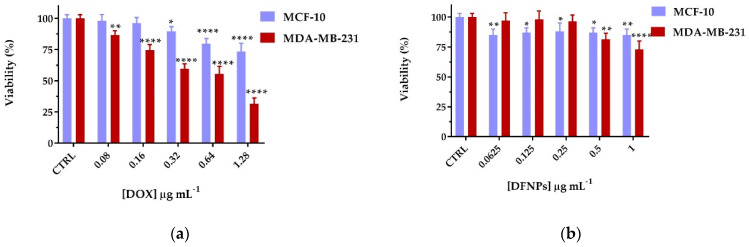
Viability percentages of (**a**) free DOX and (**b**) DFNPs on MCF-10A and MDA-MB-231 cell lines after 72 h. * *p* < 0.05, ** *p* < 0.01, and **** *p* < 0.0001 vs. each control (two-way ANOVA followed by Tukey’s post-test; data are expressed as mean ± SD of three independent experiments).

The effect of DOX, DFNPs, and DOX@DFNPs on cell proliferation was evaluated in non-tumorigenic epithelial cells (MCF-10A) and human breast adenocarcinoma cells (MDA-MB-231) after 72 h incubation. At first, the cytotoxicity of DOX or DFNPs in both cell lines was evaluated in the 0.08–1.28 μg mL^−1^ and 0.625–1.0 mg mL^−1^ concentration ranges, respectively. For free DOX, negligible cytotoxic effects were recorded on healthy cells at all the tested concentrations, while in cancer cells, a progressive decrease in cell survival in response to increasing concentrations of drug was observed, with a half-maximal inhibitory concentration (IC_50_) value of 0.58 μg mL^−1^ (Figure 8a).

Similarly, DFNPs did not exert any toxic effect on MCF-10A cells, while in MDA-MB-231 an inhibition of cell viability at a concentration up to 0.5 mg mL^−1^ was observed, as a confirmation of the safety of the proposed vehicle and the higher affinity towards tumor cells overexpressing the folate receptors.

**Figure 9:**
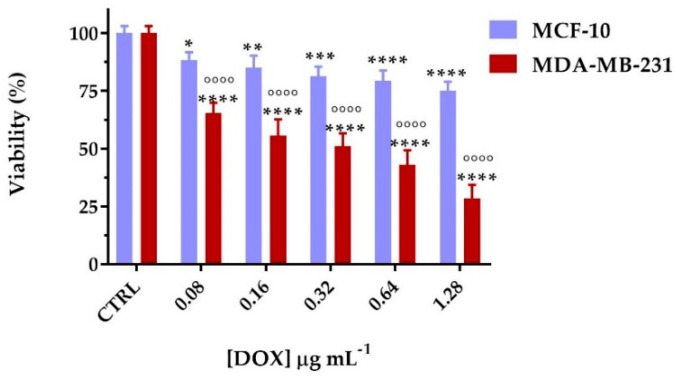
Cell viability of MCF-10A and MDA-MB-231 cells treated with DOX@DFNPs (drug concentration ranging from 0.08 to 1.28 µg mL^−1^) after 72 h of culture. * *p* < 0.05, ** *p* < 0.01, *** *p* < 0.001, and **** *p* < 0.0001 vs. each control (CTRL); °°°° *p* < 0.0001 vs. the same drug equivalent concentration on MCF-10A (two-way ANOVA followed by Tukey’s post-test; data are expressed as mean ± SD of three independent experiments).

Thus, the viability experiments with loaded nanoparticles (DOX@DFNPs) were performed fixing the nanoparticle concentration at 0.125 mg mL^−1^ to ensure both the effective formation of nanoparticles (as per CAC value of the conjugate) and negligible cytotoxic effects, and varying the DOX content according to the dose-response curve of free DOX. As depicted in Figure 9, nanoparticles retain their safety characteristics on healthy cells even when loaded with the cytotoxic drug, with cell death percentages not exceeding 20% at each tested concentration. However, when tested on MDA-MB-231, a dose-dependent decreasing of cell viability was recorded, with a significant increase of DOX activity at low drug concentration (*p* < 0.001 for 0.08 and 0.16 μg mL^−1^), an IC_50_ value almost halved compared to free DOX (0.30), due to a more efficient cellular internalization in cancer cells overexpressing the folate receptors (Figure 9).

**Figure 10:**
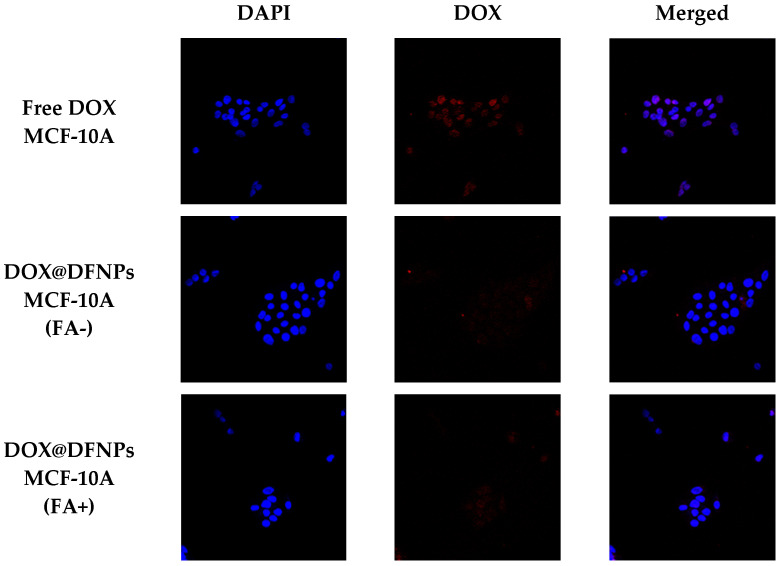

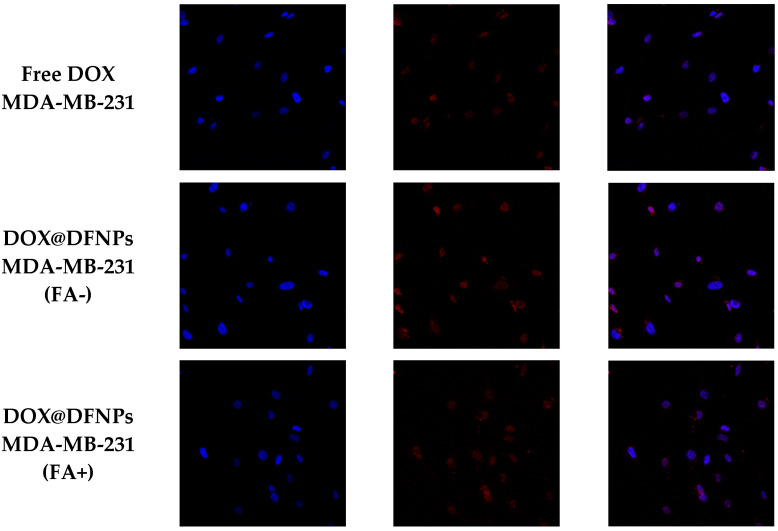
Confocal fluorescence images (40×) showing intracellular DOX or DOX@DFNPs (red fluorescence) and nuclear DAPI (blue signal) in MDA-MB-231 and MCF-10A cells. In (FA+) images, FA receptors were blocked by 1 h pretreatment with free FA, whereas (FA-) images were acquired in the absence of this receptor ligand.

**Figure 11:**
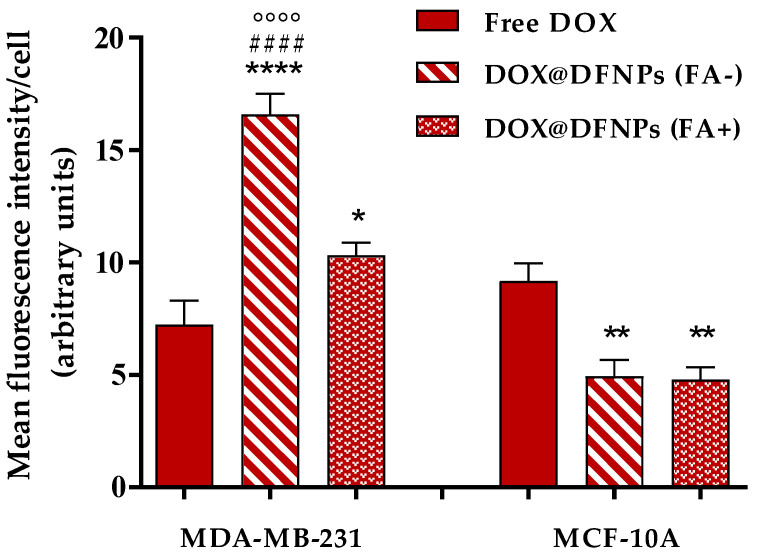
Quantitative analysis of red fluorescence intensity in MDA-MB-231 and MCF-10A cells (blocked-FA+ or unblocked-FA- receptors) exposed to DOX@DFNPs. Intensity of free DOX treatments was inserted as control. **** *p* < 0.0001 vs. free DOX; * *p* < 0.05 vs. free DOX; ** *p* < 0.01 vs. free DOX; ^####^ *p* < 0.0001 vs. DOX@DFNPs (FA+); °°°° *p* < 0.0001 vs. MCF-10A cells (FA-). (One-way ANOVA followed by Tukey’s post-test; data are expressed as mean ± SD of three independent experiments).

It is widely reported that free DOX is internalized via passive diffusion, while DOX@DFNPs are expected to enter the cells via endocytosis mediated by folate receptors, preventing the effluxion of pump-associated drug activity. The improved internalization of DOX after nanoparticle encapsulation was confirmed by cell uptake experiments. The exposure of the cancer cells to DOX@DFNPs results in a significantly enhanced fluorescent signal as compared to free DOX treatment (Figure 10), demonstrating the in vitro effectiveness of the proposed system. Furthermore, in order to assess the folate-targeting effect, both cell lines were pretreated with free FA before incubation with the nanoparticle formulation to block the folate receptors. As expected, a lower fluorescence signal was detected when the cancer cells were pretreated with free FA (Figure 11), as a demonstration of the receptor-mediated nanoparticles internalization. In addition, the lower expression of folate receptors in healthy cells was responsible for a reduced uptake of DOX@DFNPs compared to free DOX and to MDA-MB-231, with the FA pretreatment not affecting this behavior.

**Figure 12:**
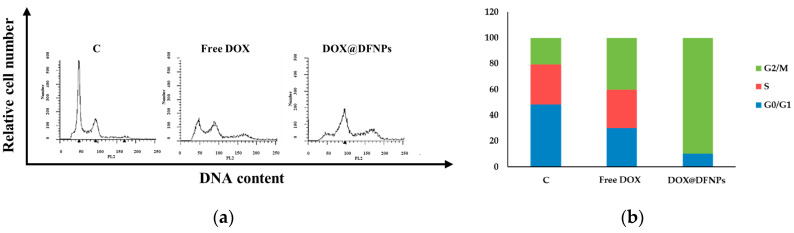
Effects of free DOX and DOX@DFNPs on cell cycle distribution in breast cancer cells. (**a**) Flow cytometry analysis of the cycle profile of breast cancer cells. MDA-MB-231 treated with 0.32 μg mL^−1^ free DOX or DOX@DFNPs for 48 h, stained with propidium iodide (PI) and analyzed on a FACS flow cytometer. (**b**) Quantitative analysis of percentage gated cell at G_0_/G_1_, S, and G_2_/M phases. C = control.

To better define the effect of free DOX and DOX@DFNPs in MDA-MB-231 cells, and to corroborate the above results, cell cycle analysis was performed. The data showed in Figure 12 demonstrated that free DOX decreased the G_0_/G_1_ phase and increased the G_2_/M phase, compared to control cells [26].

In cells treated with DOX@DFNPs, compared to free DOX, a more marked reduction of the G_0_/G_1_ phase, as well as a higher increase of G_2_/M phase, was observed.

Thus, the DOX@DFNPs caused the block of cell cycle more efficiently than free DOX in the G_2_/M phase.

## 4. Conclusions

DOX-loaded nanoparticles for targeted drug delivery were prepared by combining two self-assembling DEX conjugates with targeting (DEXFA) and pH/redox responsive (DEXssPEGCOOH) activity, respectively. The nanoparticles, with mean diameter of 50 nm and active targeting towards cancer cells via folate receptors, were found to be stable at physiological pH, while being destabilized at acidic pH and GSH concentration mimicking the intracellular environment, thus modulating the drug release profiles. Cell viability experiments on both healthy and cancer cells demonstrated the efficacy of the proposed system, with DOX@DFNPs showing a higher cytotoxic effect in MDA-MB-231 cells than in MCF-10A with respect to the free DOX. Moreover, cell uptake experiments confirmed the significant role of the nanoparticles in the vectorization of drug in cancer cells, demonstrating that this drug delivery system increased the effect on cell cycle arrest. Thus, this work proposed a simple and safe strategy to prepare new multifunctional DOX delivery systems with targeting and stimuli-responsive activity, with incoming in vivo studies being designed to further investigate the potential applications.

## Data Availability

Not applicable.

## Supplementary Materials

The following Supplementary Materials are available online at https://www.mdpi.com/article/10.3390/nano11051108/s1: Figure S1: 2D-HSCQ spectrum of DEXcys, Figure S2: 2D-HSCQ spectrum of DEXssPEGCOOH, Figure S3: 2D-HSCQ spectrum of DEXFA, Figure S4: DOSY spectrum of DEX with fitting details, Figure S5: DOSY spectrum of DEXcys with fitting details, Figure S6: DOSY spectrum of DEXssPEGCOOH with fitting details.
