# Supplementary material for: Combining Dextran Conjugates with Stimuli-Responsive and Folate-Targeting Activity: A New Class of Multifunctional Nanoparticles for Cancer Therapy
Source: Nanomaterials (Basel). 2021 Apr 25;11(5):1108. doi: 10.3390/nano11051108 (PMC8145397; doi:10.3390/nano11051108)
Supplement: Supplementary file 1 [file nanomaterials-11-01108-s001.zip › nanomaterials-1184572-supplementary.pdf]

Supplementary Materials

Combining Dextran conjugates with stimuli-responsive and folate-targeting activity: a new class of multifunctional nanoparticles for cancer therapy

Manuela Curcio[1], Alessandro Paolì[1], Giuseppe Cirillo[1], Sebastiano Di Pietro[2], Francesca Giordano[1], Loredana Mauro[1], Valeria Di Bussolo[2], Fiore Pasquale Nicoletta[1*], Francesca Iemma [1]

[1] Department of Pharmacy, Health and Nutritional Sciences, University of Calabria, 87036 Rende, Italy;
[2] Department of Pharmacy, University of Pisa, Via Bonanno Pisano 6, Pisa, 56126, Italy.
* Correspondence: fiore.nicoletta@unical.it; Tel.: 0039 0984 493194

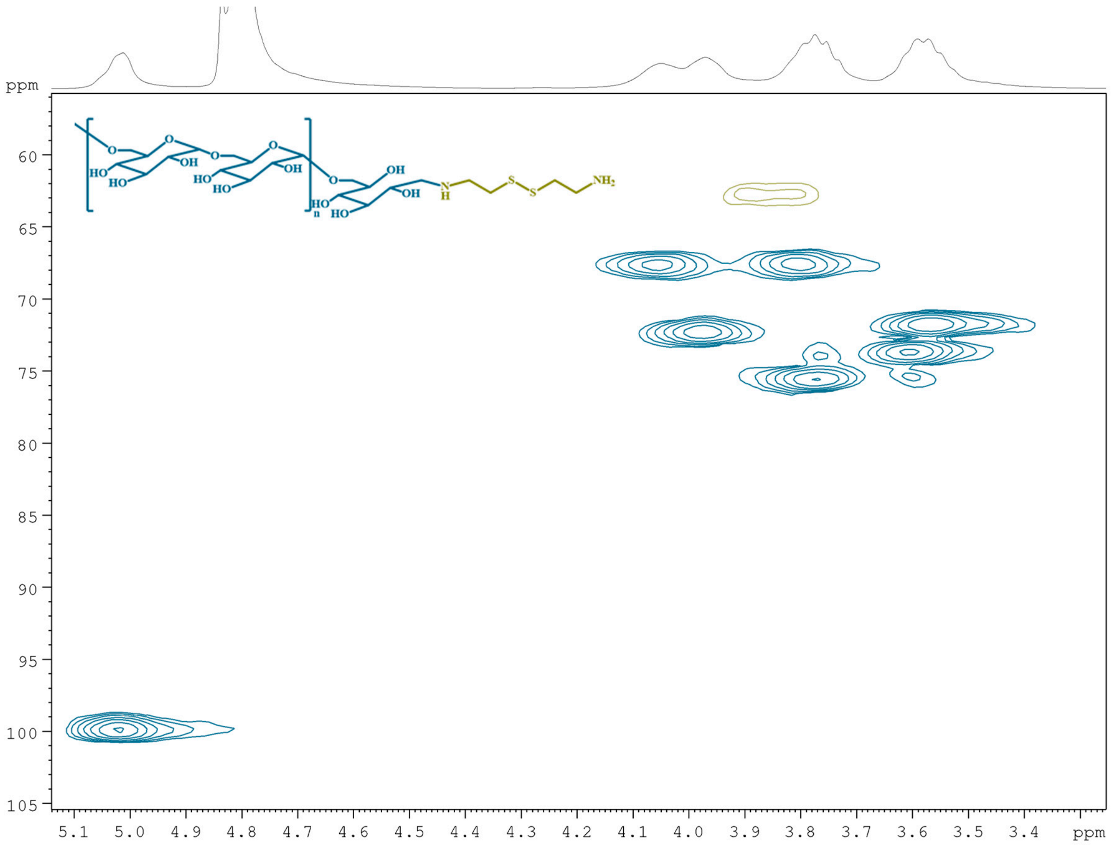

Figure S1. 2D-HSCQ spectrum of DEXcys.

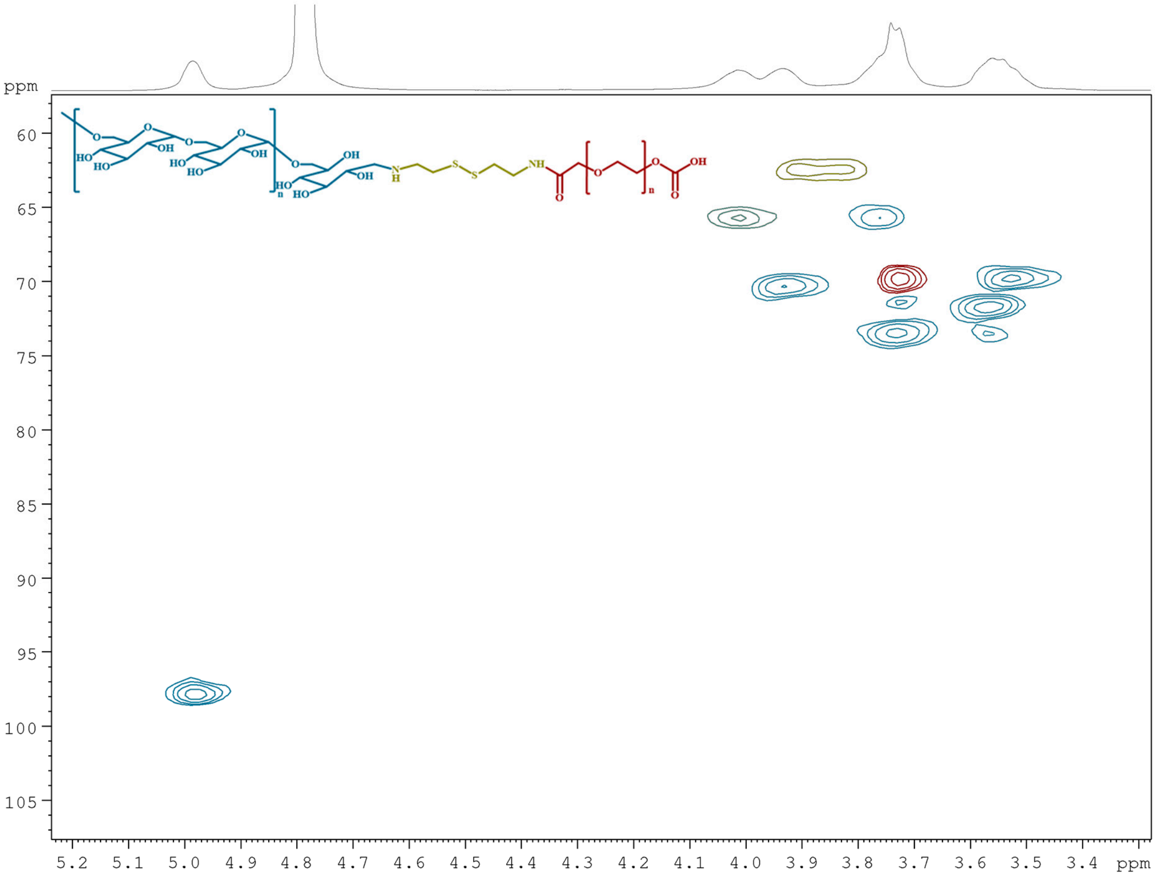

Figure S2. 2D-HSCQ spectrum of DEXssPEGCOOH.

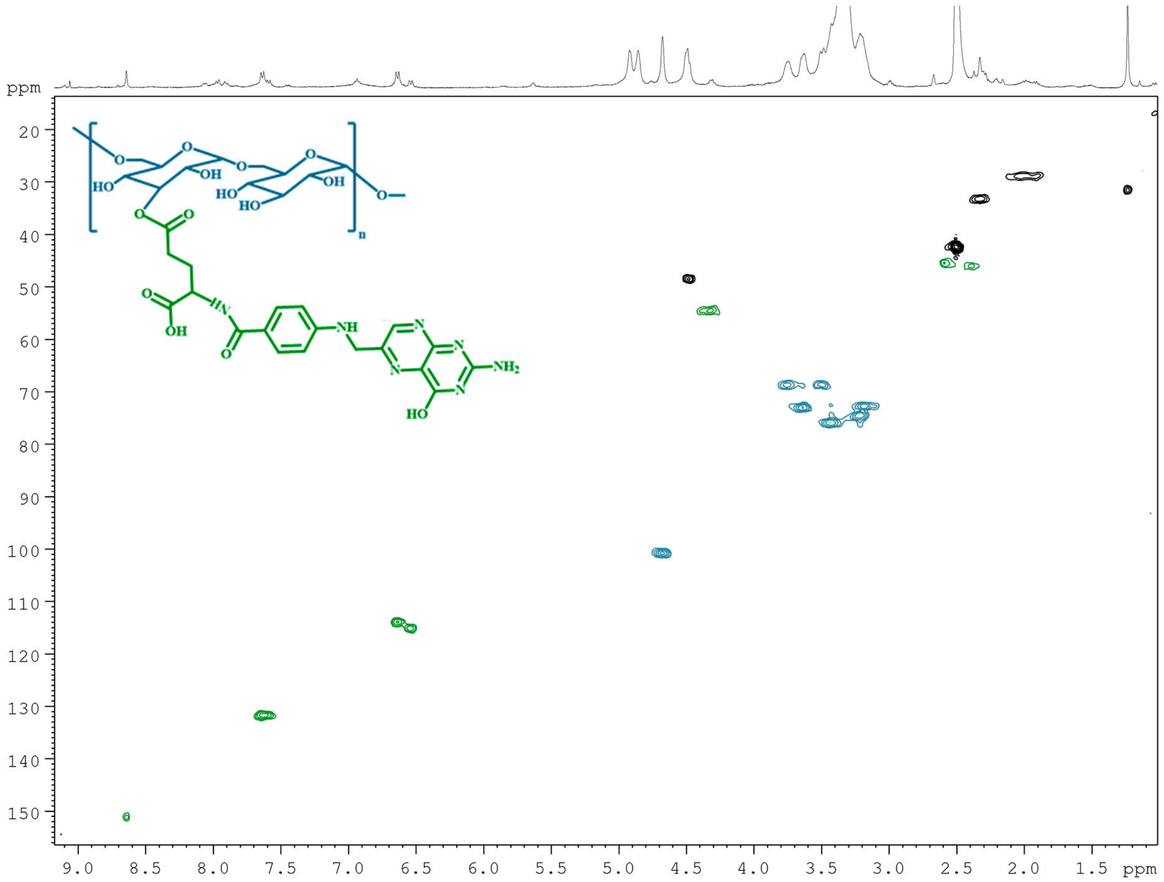

Figure S3. 2D-HSCQ spectrum of DEXFA.

^{1}H-DOSY analyses

^{1}H-DOSY spectra were recorded on a Bruker Avance III 400 MHz at 298 K. Briefly, 0.7 mg samples were dissolved in 0.6 ml of a D_2O:DMSOd_6 (2:1 v/v) mixture. The DOSY pulse sequence adopted is the ledbpgp2s, LED-bipolar gradients pulse sequence. The decay curves were obtained through 16 step magnetic field gradient pulse acquisition in a range from 1 to 52 G/cm All the parameters for the three experiments were kept identical: 60 scans, δ = 2 ms, Δ = 285 ms and g = 26752 rad/(s*Gauss). The results were elaborated with Dynamic Center, a routine of Bruker's TopSpin software. The Steiskal–Tanner analytic function, which approximates the dependence of the spin echo signal amplitude on the parameters of a magnetic field gradient pulse, was used

$$I(\delta, \Delta) = I_0 \exp[-D\gamma 2g2\delta 2(\Delta - \delta/3)]$$

where I and I_0 are the spin echo signal amplitudes in the presence and in the absence of a magnetic field gradient pulse, respectively; Δ is the diffusion time, ms; δ is the duration of a gradient pulse, ms; γ is the gyromagnetic ratio of the given nucleus, Hz/T; and g is the magnetic field gradient, T/m.

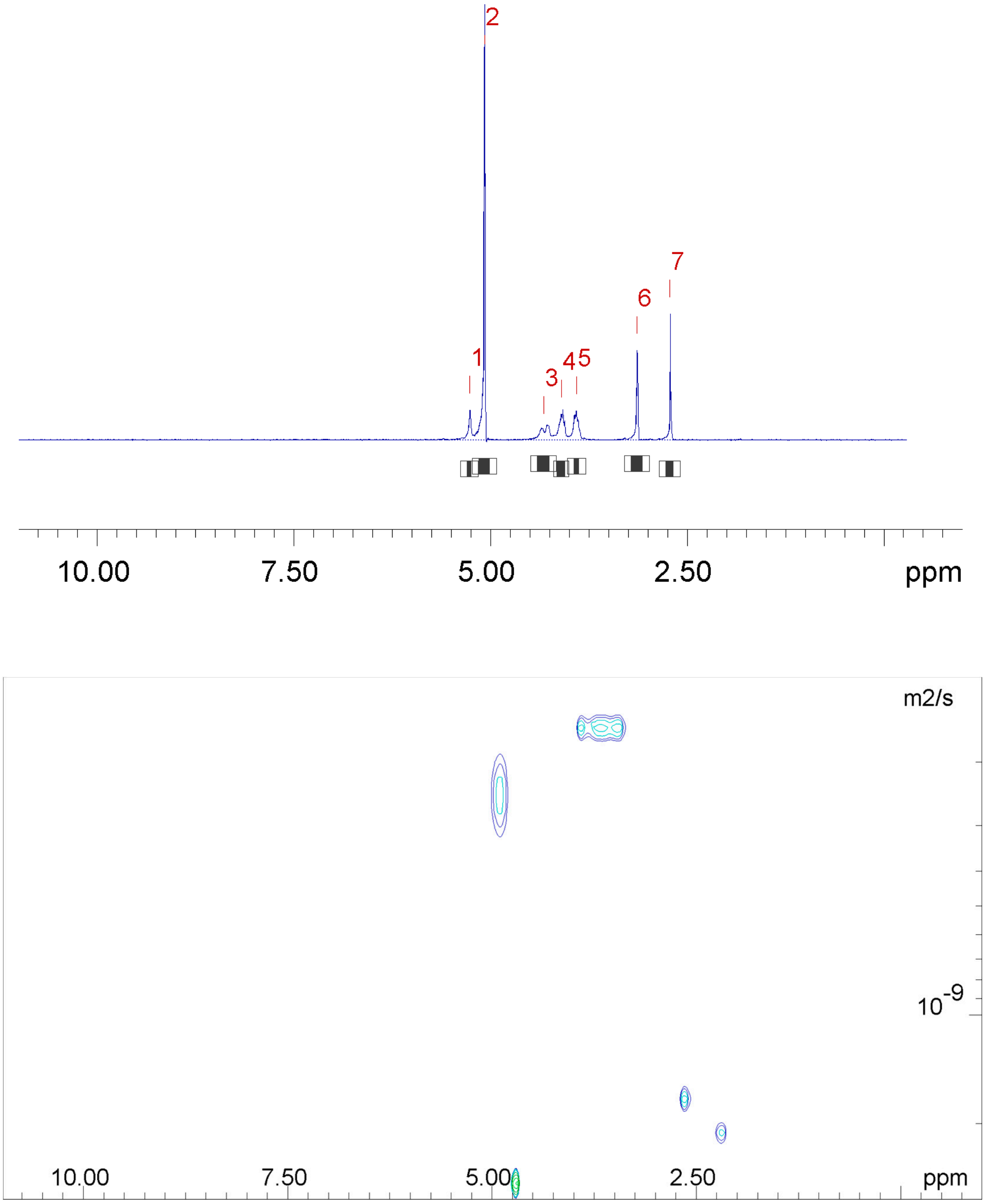

Peak name	F2 [ppm]	Io	error	D [m2/s]	error	fitInfo
1	4.902	6.20e+08	6.667e+07	2.52e-10	6.389e-11	Done
2	4.701	4.34e+09	1.574e+07	3.01e-09	2.710e-11	Done
3	3.902	8.93e+08	6.084e+06	1.56e-10	2.509e-12	Done
4	3.667	9.64e+08	8.241e+06	1.60e-10	3.226e-12	Done
5	3.460	9.49e+08	4.143e+06	1.60e-10	1.649e-12	Done
6	2.645	9.52e+08	5.988e+06	1.62e-09	2.483e-11	Done
7	2.200	6.90e+08	5.477e+06	2.01e-09	3.924e-11	Done

Figure S4. DOSY spectrum of DEX with fitting details.

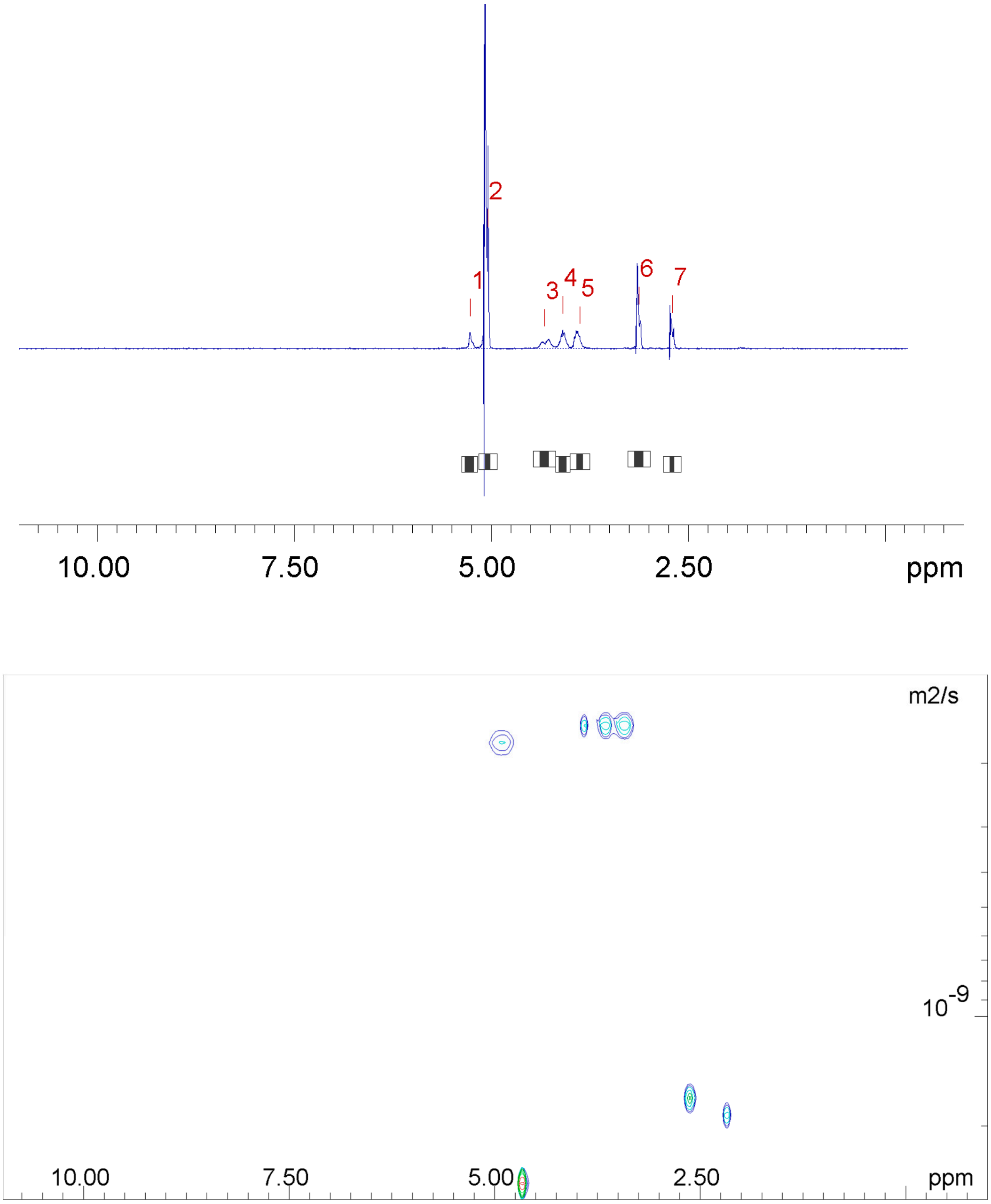

Peak name	F2 [ppm]	Io	error	D [m2/s]	error	fitInfo
1	4.909	2.64e+08	9.791e+06	1.68e-10	1.472e-11	Done
2	4.663	4.66e+09	9.364e+06	2.97e-09	1.480e-11	Done
3	3.904	4.29e+08	5.943e+06	1.52e-10	4.999e-12	Done
4	3.656	5.07e+08	8.678e+06	1.53e-10	6.205e-12	Done
5	3.423	5.40e+08	7.288e+06	1.52e-10	4.863e-12	Done
6	2.625	1.01e+09	5.501e+06	1.60e-09	2.129e-11	Done
7	2.176	4.90e+08	3.833e+06	1.90e-09	3.643e-11	Done

Figure S5. DOSY spectrum of DEXcys with fitting details.

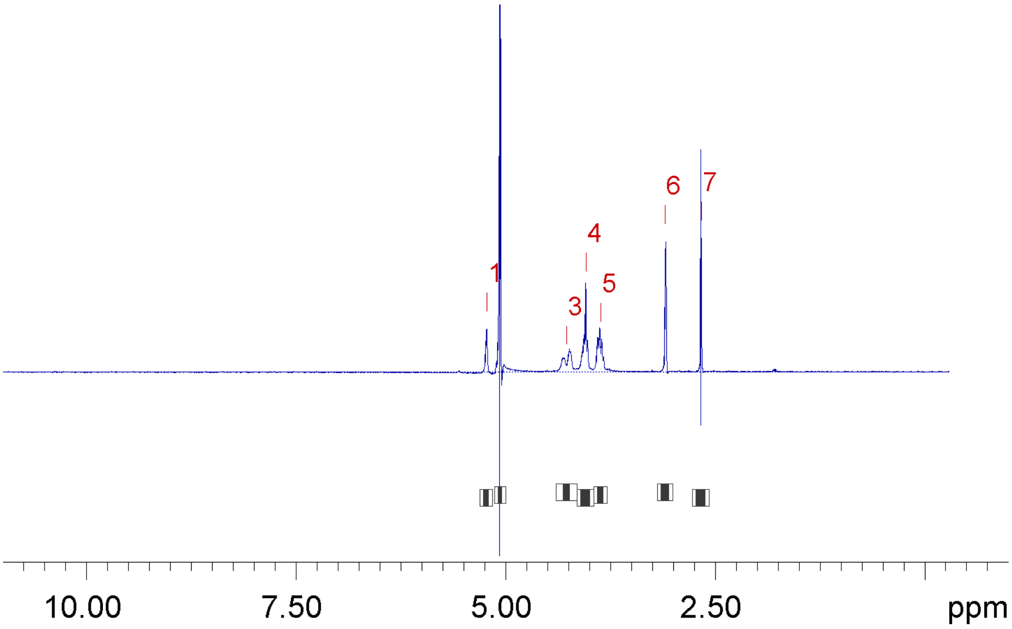

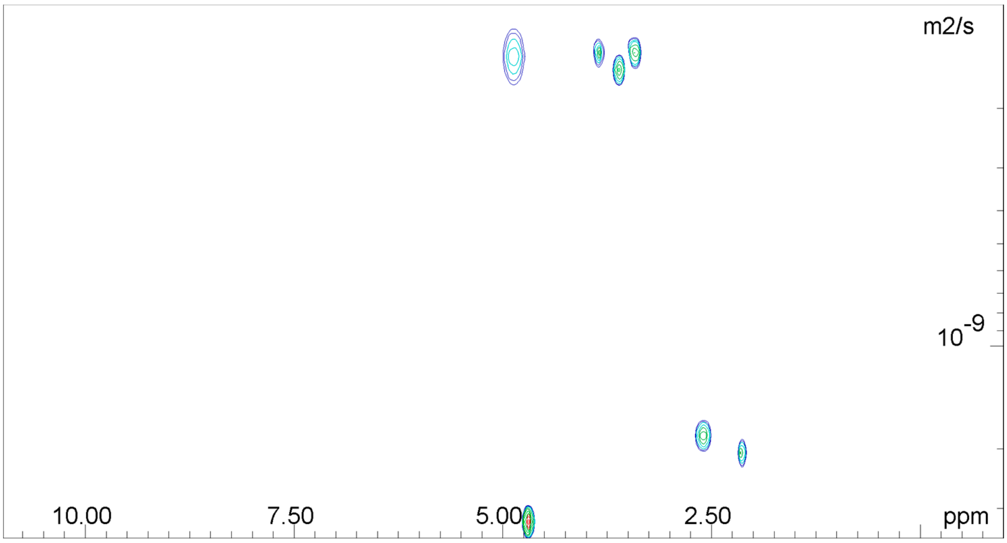

Peak name	F2 [ppm]	Io	error	D [m2/s]	error	fitInfo
1	4.864	1.54e+08	8.736e+06	1.34e-10	1.812e-11	Done
2	4.690	2.44e+09	7.150e+06	3.44e-09	2.517e-11	Done
3	3.849	3.03e+08	3.710e+06	1.32e-10	4.620e-12	Done
4	3.605	4.41e+08	7.907e+06	1.45e-10	7.560e-12	Done
5	3.421	3.73e+08	2.667e+06	1.33e-10	2.627e-12	Done
6	2.601	3.32e+08	2.741e+06	1.77e-09	3.576e-11	Done
7	2.146	2.69e+08	1.805e+06	2.06e-09	3.383e-11	Done

Figure S6. DOSY spectrum of DEXssPEGCOOH with fitting details.
